# Maltreatment and Dental Trauma in Preschool Children: A Cohort Study

**DOI:** 10.1111/edt.70046

**Published:** 2025-12-26

**Authors:** Renata Uliana Posser, Francine dos Santos Costa, Flávio Fernando Demarco, Fernando Barros, Joseph Murray, Marina Sousa Azevedo

**Affiliations:** ^1^ Graduate Program in Dentistry Federal University of Pelotas Pelotas RS Brazil; ^2^ Postgraduate Program in Epidemiology Federal University of Pelotas Pelotas RS Brazil; ^3^ Human Development and Violence Research Centre, Federal University of Pelotas Pelotas RS Brazil

**Keywords:** child abuse, child maltreatment, oral health, tooth injuries

## Abstract

**Background:**

Child maltreatment (CM) has been associated with adverse oral health outcomes during childhood. However, few studies have investigated the relationship between CM and dental trauma (DT) in the primary dentition.

**Aim:**

To evaluate the association between CM and DT at 4 years of age.

**Design:**

The sample comprised 4275 children from the 2015 Pelotas Birth Cohort Study, southern Brazil. Overall CM from birth to age 4, as well as specific types of maltreatment (“physical abuse,” “sexual abuse,” “emotional abuse,” “neglect,” and “custodial interference”), were assessed using the Juvenile Victimization Questionnaire, completed by mothers when the children were 4 years old. DT was assessed at age 4 using the criteria of the UK Child Health Survey (1993). Bivariate analyses were conducted using the chi‐square test, and crude and adjusted analyses were performed using Poisson regression models. Confounding variables with *p* < 0.250 in the crude analysis were included in the adjusted models.

**Results:**

Of the 3645 children evaluated, 11.4% had been exposed to some form of CM, and 20.2% presented with DT. In the adjusted analysis, no statistically significant association was found between DT and overall or specific types of CM.

**Conclusions:**

In this large‐sample study, no association was found between DT in the primary dentition and CM.

## 1 Introduction

Child maltreatment (CM) is defined as any act or omission by parents or caregivers that results in physical, sexual, or psychological harm or neglect of a child [1]. Exposure to CM has been linked to a wide range of adverse health outcomes across the lifespan, including risk behaviors, psychiatric disorders, and chronic diseases [2, 3, 4, 5, 6, 7, 8]. It may also serve as an important predictor of poor oral health throughout life [9, 10]. Children with a history of maltreatment have been reported to have lower levels of oral health–related quality of life [11, 12].

Research indicates that 50%–75% of child abuse cases involve trauma to the mouth, face, and head [13, 14, 15]. The high frequency of injuries in these regions is likely related to their exposure and accessibility. Moreover, the head is a highly symbolic part of the body, and the oral cavity is often a central target of abuse due to its crucial roles in communication and nutrition [13, 14, 15, 16].

Although research on CM and oral health has advanced, limited evidence is available regarding its association with dental trauma (DT), particularly in the primary dentition. Most studies examining the relationship between DT and CM have relied on convenience samples (e.g., hospitals, forensic clinics), which may introduce selection bias.

Research suggests that DT may serve as a potential marker for violence against children [17, 18]. However, no studies to date have examined the association between CM and DT in the primary dentition. Therefore, the aim of this study was to investigate this association using data from a birth cohort of young children in the city of Pelotas, Brazil.

## 2 Methodology

This study was reported in accordance with the STROBE (Strengthening Reporting of Observational Studies in Epidemiology) [19] guidelines for observational studies.

### 2.1 Study Design

This study is part of the 2015 Pelotas Birth Cohort. In 2015, all live births to mothers residing in the urban area of the city were eligible for inclusion. Additionally, a neighborhood within the municipality that had previously been classified as rural, as well as the Jardim América neighborhood in the municipality of Capão do Leão, were included to ensure comparability with previous studies' cohort studies. Of the 5598 children born in Pelotas in 2015, 4387 met the eligibility criteria. Among these, 54 were stillbirths, 51 families refused participation, and seven were lost to follow‐up, resulting in a final baseline sample of 4275 participants. In addition to the perinatal assessment conducted in 2015, four follow‐up visits were carried out at 3 months (*n* = 4110), 12 months (*n* = 4018), 24 months (*n* = 4014), and 4 years (*n* = 4010). Further details on the 2015 Pelotas Birth Cohort, including information on attrition and refusals, are available in a previous publication [20]. In the 48‐month oral health follow‐up survey, 217 participants were classified as lost to follow‐up because oral health assessments could not be conducted. These cases included interviews carried out by telephone or Skype, at home (when no oral health team was available), or in another city. Therefore, 3792 children were invited to take part in the oral health survey. In total, 3654 clinical examinations were completed, while 48 mothers and 91 children declined to participate.

The sample size (*n* = 3645) refers to participants for whom data on DT were collected during the clinical examination. The number of participants assessed for other variables may vary depending on the specific clinical condition evaluated—for instance, in cases where a child did not allow the examination to be fully completed.

### 2.2 Data Collection

In this study, we used sociodemographic data collected at birth (perinatal assessment) and, at the 4‐year follow‐up, information on oral health, CM, and sociodemographic characteristics. Perinatal data were collected in 2015 at six maternity hospitals in Pelotas, RS, Brazil, by a team of trained and calibrated interviewers and examiners. At the 4‐year follow‐up, data collection took place at the medical clinic of the Epidemiology Center of the Federal University of Pelotas, conducted by a trained and calibrated team. For families unable to attend the research center, home visits were carried out to complete the examinations.

### 2.3 Main Exposure Variables

The exposure variables in this study were related to the occurrence of CM at 4 years of age. These variables were assessed using the Juvenile Victimization Questionnaire, 2nd edition, Screener Sum Version, Caregiver Lifetime Form (JVQ‐R2) [21].

This instrument is used internationally, and numerous studies have demonstrated the reliability and validity of its responses [21]. The mothers who accompanied the children in this study were invited to take part in a private interview to complete the questionnaire, assisted by a trained interviewer.

Based on this questionnaire, the following types of lifetime maltreatment were assessed using the CM Module of the JVQ‐R2, which includes a single item for each: physical abuse, emotional abuse, neglect, and family abduction/interference with custody; sexual assault by a known adult was also assessed using an item from the Sexual Victimization Module. Each question (Table S1) inquired about lifetime victimization (yes/no).

In addition to analyzing individual types of maltreatment, an indicator of any CM was defined as having ever experienced at least one of the five types of victimization listed above. The occurrence of child abuse was recorded for each “yes” response. Scores for the child abuse domain ranged from 0 to 5, with a score of 0 indicating no experience of abuse and scores of 1–5 indicating exposure to one or more types of maltreatment.

### 2.4 Outcome

The outcome of this study was DT in the anterior primary dentition, assessed at 4 years of age. DT was evaluated according to the criteria established by the UK Child Health Survey classification system (1993) [22], which considers damage to the upper and lower incisors. Assessments were conducted through a clinical examination as part of the oral health January and November 2019. This classification system has also been used in previous surveys of other Pelotas cohorts, ensuring consistency and comparability across studies. The clinical oral health examination at age 4 years was performed on 3654 children, of whom 3645 underwent the assessment for DT, representing 90.9% of the cohort segment.

Data were collected for each anterior tooth and classified as “without trauma,” “enamel fracture,” “enamel and dentin fracture,” “any fracture with signs or symptoms of pulpal involvement,” “no fracture but with signs or symptoms of pulpal involvement,” “tooth lost due to trauma,” or “other damage” when types of trauma not previously listed occurred. For analytical purposes, DT was categorized as absent (without trauma) or present (any type of DT). DT was also evaluated according to severity and classified into enamel fracture, enamel and/or dentin fracture, pulpal involvement, and avulsion.

The oral health examination was conducted by dentists who had been previously trained and calibrated. Theoretical training lasted 8 h, followed by practical training on 12 four‐year‐old children who were not part of the cohort. The minimum kappa index accepted for this study was 0.60, and the mean kappa obtained for DT was 0.76. For occlusion, which was included as a covariate in the statistical analysis, the mean kappa was 0.71. All examinations adhered to WHO biosafety guidelines. Personal protective equipment, a portable artificial light, a mouth mirror, and a standard NIDR periodontal probe were used during the oral assessments. Data were recorded by the examiner on a clinical examination form specifically developed for the survey.

### 2.5 Covariates

The sociodemographic variables collected in the perinatal study and included in the analysis were maternal age at the child's birth (in complete years), categorized as < 20 years (adolescents) and ≥ 20 years [23] (adults and the child's sex (male/female)). Variables collected at the 4‐year follow‐up included maternal marital status, assessed with the question, “Do you have a husband or partner?” (yes/no); presence of siblings (yes/no); the number of people living in the household, including the child, was collected as a continuous variable and then dichotomized (≤ 4/> 4) based on the median value observed in the study sample; maternal education, categorized by years of formal education completed (0–4, 5–8, 9–11, or ≥ 12 years); and family income in the month prior to the interview, categorized as ≤ 1, 1.1–3.0, 3.1–6.0, 6.1–10.0, or > 10 minimum wages. Oral health variables used for confounding adjustment included occlusal deviations, overjet, and overbite. These were assessed according to the criteria established by Foster and Hamilton [24] during the 4‐year follow‐up clinical examination. Overjet was recorded as normal, increased, end‐to‐end, or anterior crossbite and categorized for analysis as (i) normal/anterior crossbite/end‐to‐end or (ii) increased. Overbite was recorded as normal, reduced, open, or deep and categorized as (i) without open bite or (ii) with open bite.

### 2.6 Statistical Analysis

Descriptive analyses (absolute and relative frequencies) were conducted for all study variables. Associations were evaluated using the chi‐square test, with a significance level of 5%. Crude and adjusted analyses were performed to examine the relationship between the exposure variables (CM and its subtypes) and the outcome (DT) using Poisson regression models to estimate prevalence ratios and 95% confidence intervals. Variables with *p* < 0.250 in the crude analysis were included in the adjusted models (sex, overjet, and overbite). Each model was adjusted independently, with only one type of maltreatment per model. Variables with *p* < 0.05 in the adjusted analysis were considered statistically significant. The association between DT severity and CM experience was also assessed. All statistical analyses were performed using Stata version 17.0.

### 2.7 Ethical Aspects

The cohort study was approved by the Ethics Committee of the School of Physical Education, Federal University of Pelotas (CAAE registration number: 26746414.5.0000.5313). The 48‐month psychosocial assessments, including evaluations of exposure to violence, were approved by the Ethics Committee of the Faculty of Medicine, Federal University of Pelotas (CAAE registration number: 03837318.6.0000.5317). Written informed consent was obtained from the parents or legal guardians at each follow‐up visit.

## 3 Results

In this sample (*n* = 3645), the prevalence of DT was 20.2%. Table 1 presents the characteristics of the study population according to the presence or absence of DT, including sociodemographic variables (age, maternal education, and marital status, presence of siblings), occlusal deviations (overjet and overbite), and CM (physical abuse, psychological abuse, neglect, custodial interference, and sexual abuse). DT was significantly associated with sex and overjet, being more frequent in male children (*p* = 0.001) and in those with increased overjet (*p* < 0.001). The overall prevalence of reported CM was 11.4%. Physical abuse, psychological abuse, neglect, and custodial interference were reported by caregivers in 1.9%, 7.9%, 2.0%, and 2.2% of the sample, respectively; sexual abuse was not reported. Among children who had experienced maltreatment, the prevalence of DT was higher than in those without reported exposure. However, no statistically significant association was observed between DT and overall exposure to CM (as assessed by the JVQ) or any specific type of abuse.

**TABLE 1:** Association between trauma, child abuse, sociodemographic variables, and occlusion in the sample of live births from the 2015 Cohort (2015–2019), Pelotas/Brazil (*n* = 3645).

Independent variables	Presence of dental trauma			
	*N* total (%)	Yes (%)	No (%)	*p*
Gender (*n* = 3645*)				0.001
Female Male	1803 (49.5) 1842 (50.5)	323 (17.9) 412 (22.4)	1480 (82.1) 1430 (77.6)	
Maternal age in years (*n* = 3644*)				0.237
< 20 ≥ 20	526 (14.4) 3119 (85.6)	430 (81.7) 2480 (79.5)	96 (18.3) 639 (20.5)	
Number of people living in the house (*n* = 3643*)				0.248
≤ 4 > 4	2664 (73.1) 980 (26.9)	549 (20.6) 185 (18.9)	2115 (79.4) 795 (81.1)	
Brothers (*n* = 3643*)				0.390
Yes No	2076 (57.0) 1567 (43.0)	428 (20.6) 305 (19.5)	1648 (79.4) 1262 (80.5)	
Mother has partner/husband (*n* = 3637*)				0.540
Yes No	2913 (80.1) 724 (19.9)	593 (20.4) 140 (19.3)	2320 (79.6) 584 (80.6)	
Maternal education in years (*n* = 3095*)				0.673
0–4 5–8 9–11 12+	136 (4.4) 866 (27.9) 946 (30.6) 1147 (37.1)	24 (17.7) 173 (20.0) 188 (19.9) 245 (21.4)	112 (82.3) 693 (80.0) 758 (80.1) 902 (78.6)	
Family income in minimum wages (1 salary = BRL 998.00) (*n* = 3605*)				0.821
≤ 1 1.1–3.0 3.1–6.0 6.1–10.0 > 10	399 (11.1) 1751 (48.6) 963 (26.0) 272 (7.5) 247 (6.8)	72 (18.1) 359 (20.5) 191 (20.4) 54 (19.9) 53 (21.5)	327 (81.9) 1392 (70.5) 745 (79.6) 218 (80.1) 194 (78.5)	
Overjet (*n* = 2357*)				< 0.001
Normal/Top to top/Back cross Increased	1543 (65.5) 814 (34.5)	260 (16.8) 194 (23.8)	1283 (83.2) 620 (76.2)	
Overbite (*n* = 3.486*)				0.108
No open bite With an open bite	2145 (61.5) 1341 (38.5)	411 (19.2) 287 (21.4)	1734 (80.8) 1054 (78.6)	
JVQ—Experience of maltreatment (*n* = 3641)				0.116
Yes No	416 (11.4) 3229 (88.6)	96 (23.1) 639 (19.8)	320 (76.9) 2590 (80.2)	
Physical abuse (*n* = 3644*)				0.316
Yes No	68 (1.9) 3576 (98.1)	17 (25.0) 718 (20.1)	51 (75.0) 2858 (79.9)	
Psychological abuse (*n* = 3644*)				0.383
Yes No	289 (7.9) 3255 (92.1)	64 (22.2) 671 (20.0)	225 (77.8) 2684 (80.0)	
Negligence (*n* = 3645)				0.623
Yes No	72 (2.0) 3573 (98.0)	16 (22.2) 719 (20.1)	56 (77.8) 2854 (79.9)	
Custodial interference (*n* = 3643*)				0.457
Yes No	81 (2.2) 3562 (97.8)	19 (23.5) 716 (20.1)	62 (76.5) 2846 (79.9)	

*Note: p* values obtained from the Chi‐square test.

* Unanswered data were considered missing in the statistical analysis.

**FIGURE 1:**
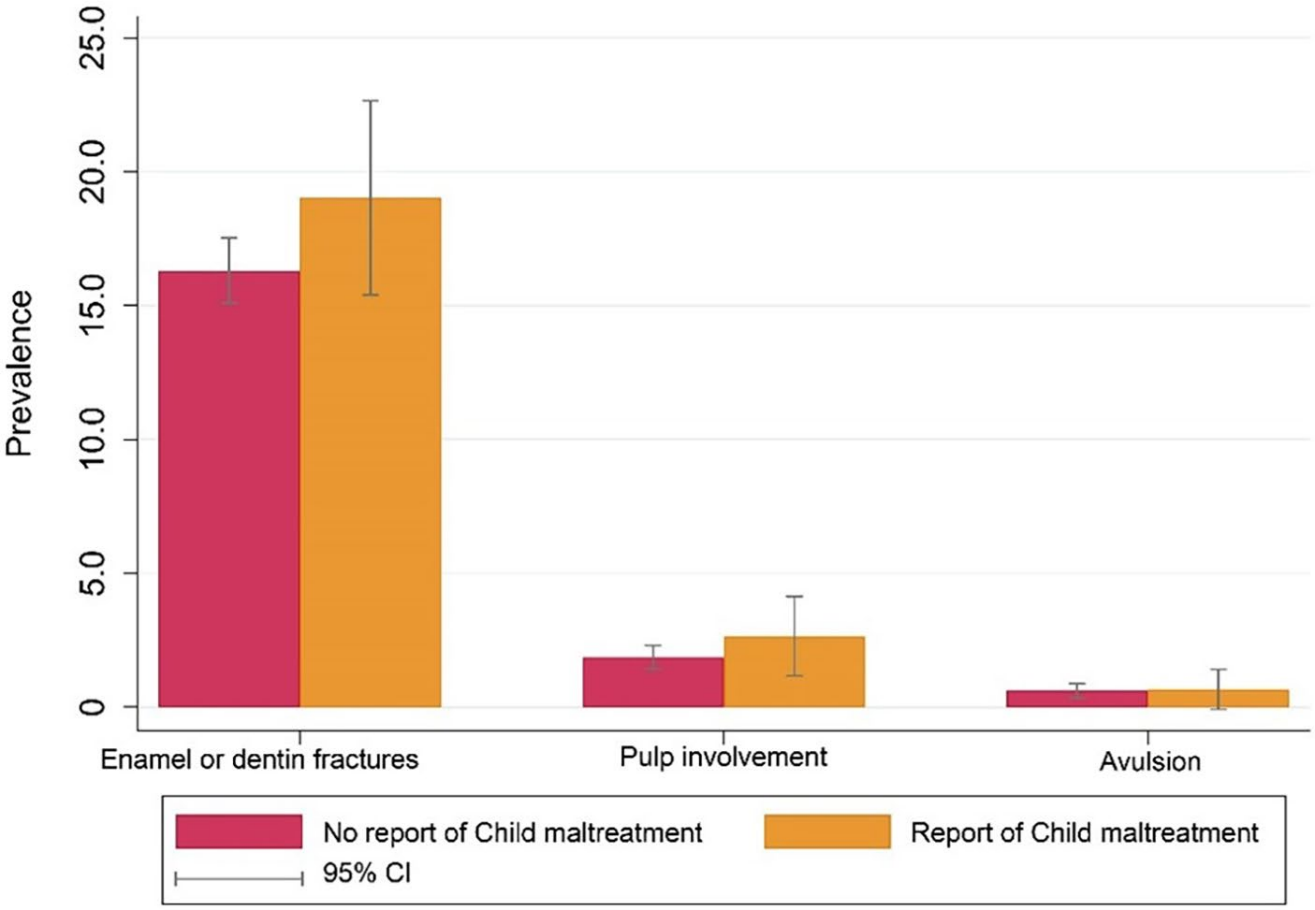
Description of the proportion of types of DT (severity) according to the CM report, Pelotas Birth Cohort, 2015.

Figure 1 illustrates the distribution of dental trauma (DT) types according to reported child maltreatment (CM). Lesions involving enamel and/or dentin were the most frequent among children with reported CM, followed by lesions with pulpal involvement. No differences in the proportion of avulsions were observed between children with and without reported CM.

Table 2 presents the results of the crude and adjusted analyses. No statistically significant association was observed between DT and any type of CM, either in the crude or adjusted models.

**TABLE 2:** Poisson regression coefficients, crude and adjusted for the association between dental trauma and child maltreatment.

	Dental trauma					
	PR (crude)	95% CI	*p*	PR (adjusted)*	95% CI	*p*
JVQ—CM experience	1.16	0.94–1.45	0.160	1.19	0.90–1.57	0.203
Physical abuse	1.24	0.78–2.02	0.372	1.23	0.63–2.38	0.531
Psychological abuse	1.11	0.85–1.44	0.436	1.21	0.87–1.66	0.251
Negligence	1.10	0.67–1.82	0.695	0.96	0.47–1.92	0.900
Custodial interference	1.16	0.74–1.85	0.507	1.14	0.66–1.99	0.622

*Note:* Variables *p* > 0.250 were not included in the analysis. Family income in minimum wages, maternal education, in years, whether the mother has a partner/husband, brothers, the number of people living in the house, and maternal age in years.

Abbreviations: 95% CI, confidence interval of 95%; PR, Prevalence ratio.

* Adjusted for gender, overbite, and overjet variables. Reference category: no reports of abuse. The models were independently adjusted, including only one exposure to maltreatment per model.

## 4 Discussion

To date, no study has evaluated the presence of DT in the primary dentition among children with reported CM. In the present study, no association was found between CM and the occurrence of DT in the primary dentition, regardless of the type of maltreatment assessed.

Previous research has demonstrated an association between DT and CM; however, most studies were conducted in convenience samples, which may introduce selection bias and influence the results [2, 25].

As mentioned above, no association was found between DT and CM in our sample. It is important to note that CM often occurs within a generally hostile environment, with reduced care and attention to many of the child's needs. Therefore, studying different forms of abuse and their intersections is essential to improve understanding of this type of violence and its impact on oral health [26]. Additionally, oral health problems, such as caries and DT, can serve as markers of broader systemic violence against children [17]. In a microsystem of family violence, where maltreatment occurs, it is unlikely that a child who experiences physical abuse will not also be exposed to other forms of violence, which can influence outcomes. For this reason, this study evaluated all types of CM to which the child may be exposed. Additionally, accidental and intentional trauma were not differentiated because both can occur in the context of CM. Intentional trauma may result from direct physical abuse, whereas accidental DT can stem from neglect.

Despite the high prevalence [1, 3], violence against children is often hidden, underreported [1] and underestimated, as prevalence estimates are typically based on data from official records, such as those from health systems or the judiciary. In addition, widespread beliefs among both adults and children to perceive violence as normal rather than as a problem requiring attention [27, 28]. Another factor that may have influenced our results is that very young children may not accurately report abuse experiences to their caregivers. Individuals begin to develop a more efficient ability to classify and organize their thoughts from around the age of 12 [29]. In this context, the guardian may be unaware of the violence experienced by the child before this age. Further studies involving this age group are important, as younger children exhibit higher victimization rates. According to reports carried out by Child Protective Services (CPS), approximately 29% of child abuse cases that occurred in the year 2020 in the USA involved victims aged ≤ 2 years of age [30]. Data from the Brazilian Yearbook of Public Health showed a 21% in child abuse cases in 2021, with the 0–4 age group experiencing the second‐highest increase in incidence [31].

Studies show a higher prevalence of traumatic injuries in the head and neck region among children who have experienced CM, with craniofacial injuries occurring in more than half of such cases [15, 31, 32, 33]. However, regarding DT, especially in deciduous teeth, the literature remains scarce. A study that evaluated the dental aspects of abused children in a hospital showed that injuries to the head, face, and neck were frequent in children who suffered some type of violence. However, despite the high number of reports of head and neck injuries, reports of oral injuries, such as DT, were low in both primary and permanent teeth [14]. Injuries to permanent teeth were twice as frequent in children with a history of abuse in a comparative study involving abused children, monitored at a reference center for CM, and students without a history of violence [34]. No studies were found in the literature that specifically addressed the relationship between CM and DT in the primary dentition.

Most studies mapping traumatic injuries in the head region among victims of abuse are based on hospital records, forensics, and institutions that provide assistance to victims [32], where there is confirmation. In most cases, however, injuries are not recorded by dental specialists, which may lead to underreporting of DT and lower prevalence estimates than the true rates.

A key strength of this study is that it is a population‐based epidemiological investigation using data from clinical examinations conducted by dentists who were properly trained and calibrated within a birth cohort. Importantly, these dentists had no prior knowledge of CM, which was assessed separately by a separate interviewer.

A limiting factor in our study is that the mother was the only informant on CM, which increases the potential for common reporting and social desirability biases, potentially resulting in underreporting. Notably, no cases of sexual abuse were identified using the JVQ instrument. This absence may be explained by social desirability bias, the mother's lack of awareness, or the child's limited understanding, particularly among very young children, who may be unable to recognize an act of sexual violence. It should also be considered that, in some cases, the mother herself may be the perpetrator of maltreatment, which could lead her to underreport or deny such events. These factors, taken together, may contribute to an underestimation of the true prevalence of CM and may also explain the absence of reported cases of sexual abuse. Another limitation is that the questionnaire was administered retrospectively, and this information may have been influenced by recall bias or the respondent's psychological functioning. Additionally, no information was available regarding the perpetrators of IPV or CM. Therefore, the results of this study cannot be generalized to other contexts due to social, economic, and cultural variability within the population. Although one of the aims of the questionnaire is to enhance correspondence with official data, positive responses to its items do not constitute a diagnosis in themselves; further individual and complementary investigations would be required to determine whether any type of violence identified through the JVQ would be subject to formal reporting. One of the advantages of using this instrument lies in its rigorous development process, which established reliability and validity for its scores. It was designed to minimize response errors and is suitable for administration to children aged 8 years and older, as well as to adolescents and caregivers. Its comprehensive approach to victimization encompasses multiple types of violence, which can support the evaluation of services for children and families, identify community needs, and assess the effectiveness of prevention or intervention programs [21].

Another limiting factor is that some types of DT, such as concussion and subluxation, may not be identified in epidemiological examinations, leading to undervaluation in this type of study. The forms of DT that can be captured in such examinations are generally limited to hard tissue injuries (enamel and dentin), missing teeth (avulsion), and sequelae such as discoloration.

## 5 Conclusion

In this large population‐based study, no association was found between DT in the primary dentition and CM. Future research should investigate this association using multiple informants and larger datasets, incorporating individual behavioral and health characteristics, as well as family and environmental contexts, to provide a more comprehensive understanding of the complex relationship between CM and oral injuries.

## Author Contributions

Each author contributed meaningfully to the development of this manuscript. Contributions encompassed the design of the study, methodological planning, data validation and investigation, provision of resources, preparation of the initial draft, and subsequent critical revision and editing. All authors reviewed and approved the final manuscript prior to submission.

## Funding

This work was supported by Wellcome Trust, Conselho Nacional de Desenvolvimento Científico e Tecnológico, Fundação de Amparo à Pesquisa do Estado do Rio Grande do Sul, Children's Pastorate, Fundação Bernard van Leern, Departamento de Ciência e Tecnologia (DECIT/Ministério da Saúde do Brasil, Instituto Todos Pela Saúde, and Celer Biotecnologia).

## Conflicts of Interest

The authors declare no conflicts of interest.

## Supporting information


**Table S1:** Child maltreatment domain questions JVQ.

## Data Availability

The data that support the findings of this study are available on request from the corresponding author. The data are not publicly available due to privacy or ethical restrictions.
