# Supplementary material for: Maltreatment and Dental Trauma in Preschool Children: A Cohort Study
Source: Dent Traumatol. 2025 Dec 26;42(4):482–9. doi: 10.1111/edt.70046 (PMC13356496; doi:10.1111/edt.70046)
Supplement: Supplementary file 1 — Table S1: Child maltreatment domain questions JVQ. [file EDT-42-482-s001.docx]

**Supporting table.** Child Maltreatment Domain Questions  JVQ.

| **Form of child maltreatment** | **Question** |
| --- | --- |
| Physical abuse | An adult hit, kicked, or physically hurt your child in any way, not including spanking your child's ass |
| Psychological abuse | Has your child ever in his/her life been scared or felt bad because adults insulted him/her, said mean things to him/her, or said they didn't want him/her? |
| Negligence | When someone is neglected, it means that the adults in their life have not taken care of them the way they should have. You may not have given them enough food, taken them to the doctor when they got sick, or made sure they had a safe place to stay. At any point in your child's life, has he/she been neglected? |
| Custodial interference | Sometimes a family disputes over where the child should live. Has one of your parents ever taken, taken or hidden from her/your child to prevent you from staying with the other parent? |
| Sexual abuse | Has any adult your child knows touched your child's nether (intimate) parts where they shouldn't have or made your child touch his/her nether (intimate) parts? Or did it happen that an adult that she / her child knew forced her / her child / to have sex? |
